# Randomised phase II study of docetaxel/cisplatin *vs* docetaxel/irinotecan in advanced non-small-cell lung cancer: a West Japan Thoracic Oncology Group Study (WJTOG9803)

**DOI:** 10.1038/sj.bjc.6601462

**Published:** 2004-01-06

**Authors:** N Yamamoto, M Fukuoka, S-I Negoro, K Nakagawa, H Saito, K Matsui, M Kawahara, H Senba, Y Takada, S Kudoh, T Nakano, N Katakami, T Sugiura, T Hoso, Y Ariyoshi

**Affiliations:** 1 Department of Medical Oncology, Kinki University School of Medicine, 377-2 Ohnohigashi, Osakasayama, Osaka 589-8511, Japan

**Keywords:** combination chemotherapy, doublets, irinotecan, cisplatin, docetaxel, non-small-cell lung cancer, carboplatin

## Abstract

Docetaxel plus cisplatin and docetaxel plus irinotecan are active and well-tolerated chemotherapy regimens for advanced non-small-cell lung cancer (NSCLC). A randomised phase II study compared their efficacy and toxicity in 108 patients with stage IIIb/IV NSCLC, who were randomised to receive docetaxel 60 mg m^−2^ and cisplatin 80 mg m^−2^ on day 1 (DC; *n*=51), or docetaxel 60 mg m^−2^ on day 8 and irinotecan 60 mg m^−2^ on day 1 and 8 (DI; *n*=57) every 3 weeks. Response rates were 37% for DC and 32% for DI patients. Median survival times and 1- and 2-year survival rates were 50 weeks (95% confidence interval: 34–78 weeks), 47 and 25% for DC, and 46 weeks (95% confidence interval: 37–54 weeks), 40 and 18% for DI, respectively. The progression-free survival time was 20 weeks (95% confidence interval: 14–25 weeks) with DC and 18 (95% confidence interval: 12–22 weeks) with DI. Significantly more DI than DC patients had grade 4 leucopenia and neutropenia (*P*<0.01); more DC patients had grade ⩾2 thrombocytopenia (*P*<0.01). Nausea and vomiting was more pronounced with DC (*P*<0.01); diarrhoea was more common with DI (*P*=0.01). Three treatment-related deaths occurred in DC patients. In conclusion, although the DI and DC regimens had different toxicity profiles, there was no significant difference in survival.

Unfortunately, non-small-cell lung cancer (NSCLC) is a member of the group of neoplastic diseases that is relatively chemoresistant. Recent meta-analyses show that cisplatin-based chemotherapy improves survival (Non-Small Cell Lung Cancer Collaborative Group, 1995), and it is considered a standard treatment for NSCLC, Most cisplatin-based regimens have substantial toxicities that require close monitoring and supportive care. Thus, there is a need to develop active and less toxic chemotherapy regimens that include new active compounds with novel mechanisms of action.

In the 1990s, several new, active therapies with single-agent response rates of 15–30% became available for NSCLC, including irinotecan, docetaxel, paclitaxel, vinorelbine, and gemcitabine. Because irinotecan and docetaxel were approved for NSCLC earlier than the other drugs in Japan, development of regimens containing irinotecan or docetaxel is more advanced. Docetaxel 60 mg m^−2^ showed good antitumour activity against advanced NSCLC (Kunitoh *et al*, 1996), and the combination of docetaxel plus cisplatin (DC) is one of the most effective regimens for advanced NSCLC (Rodriguez *et al*, 2001; Schiller *et al*, 2002). Studies in Japan included a phase II study in which DC yielded a response rate of 42% (Okamoto *et al*, 2002), and a phase III study in which DC was associated with better survival than the vindesine and cisplatin (VC) combination (Kubota *et al*, 2002).

Irinotecan demonstrated activity similar to that of VC in stage IIIb/IV NSCLC (Negoro *et al*, 2003), and significant longer overall survival time than VC in stage IV NSCLC (Fukuoka *et al*, 2000). We reported a phase I study of docetaxel plus irinotecan (DI) in patients with advanced NSCLC, in which a promising response rate of 48% and the median survival time of 48 weeks were achieved with acceptable toxicities (Masuda *et al*, 2000). Thus, DI appeared to be a promising non-cisplatin-containing regimen.

Based on the above findings, we conducted a randomised trial of DC *vs* DI in patients with advanced NSCLC to compare the respective response rates, survival data, and toxicity profiles of the two regimens. This was a multicentred phase II study.

## PATIENTS AND METHODS

### Patients

Patients enrolled in this trial had histologically or cytologically confirmed stage IIIb or IV NSCLC. Patients with stage IIIb disease who were not candidates for thoracic radiation and patients with stage IV disease were eligible if they had not received previous therapy, had measurable disease, and had a life expectancy of at least 3 months. Additional entry criteria were age ⩾20 years, performance status of 0 or 1 on the Eastern Cooperative Oncology Group (ECOG) scale, adequate bone marrow function (leucocyte count 4000–12 000 *μ*l^−1^, haemoglobin concentration ⩾9.5 g dl^−1^ platelet count ⩾100 000 *μ*l^−1^), kidney function (creatinine ⩽ upper limit of normal, 24-h creatinine clearance ⩾60 ml min^−1^), liver function (aspartate aminotransferase (AST) and alanine aminotransferase (ALT) ⩽2.0 times the upper limit of normal, total bilirubin ⩽1.5 mg dl^−1^), and pulmonary function (*P*aO_2_⩾60 torr). Patients with active concomitant or a recent (<3 years) history of any malignancy, symptomatic brain metastases, past history of drug allergy reactions, complication by interstitial pneumonia, watery diarrhoea, ileus, treatment with nonsteroidal anti-inflammatory drugs, or other serious complications, such as uncontrolled angina pectoris, myocardial infarction within 3 months, heart failure, uncontrolled diabetes mellitus or hypertension, massive pleural effusion or ascites, or serious active infection were excluded. All patients gave written informed consent, and the institutional review board for human experimentation approved the protocol.

### Study evaluations

Pretreatment studies included a complete medical history and physical examination, chest X-ray, electrocardiography, computed tomography (CT) scan of the brain and chest, CT or ultrasound examination of the abdomen, and bone scintigraphy. Blood and blood chemistry studies included complete blood cell count, liver function test, serum electrolytes, serum creatinine, and blood urea nitrogen. Chest X-ray, blood and blood chemistry analyses, and urinalysis were repeated weekly.

### Randomisation and treatment schedule

**Figure 1:**
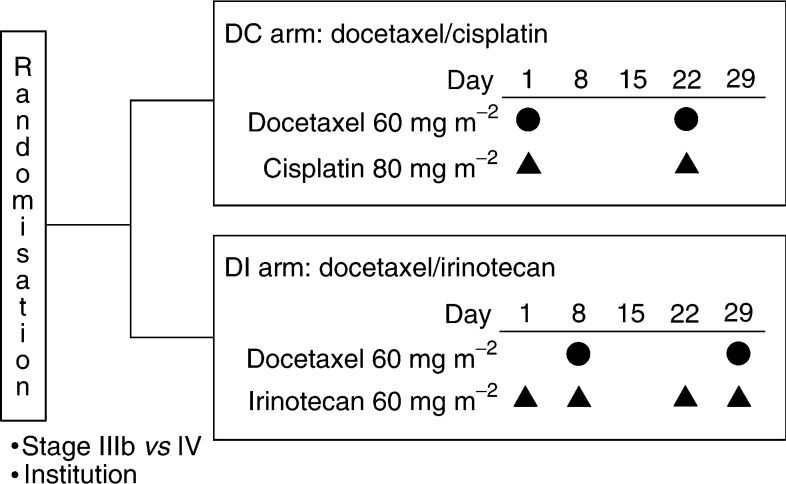
*Treatment schema*: after stratification by stage and institution, enrolled patients were randomly allocated to receive docetaxel plus cisplatin (DC) or docetaxel plus irinotecan (DI).

Patients were randomly assigned to receive the DC regimen or the DI regimen by a minimisation method using stage (IIIB/IV) and treatment institution. The DC regimen was consisting of docetaxel 60 mg m^−2^ on day 1 and cisplatin 80 mg m^−2^ on day 1, and the DI regimen was consisting of docetaxel 60 mg m^−2^ as a 60-min intravenous infusion on day 8 and irinotecan 60 mg m^−2^ as a 90-min intravenous infusion on days 1 and 8 (Figure 1

). Both regimens were repeated every 3 weeks. Participating researchers at each institution decided the amount of fluid replacement and the type of antiemetic therapy to administer. Standard antiemetic treatment in the DC arm consisted of 5-HT_3_ receptor antagonist plus 16 mg dexamethasone intravenously on day 1, before cisplatin administration. In the DI arm, standard antiemetic treatment consisted of 5-HT_3_ receptor antagonist intravenously before chemotherapy administration on days 1 and 8. Patients received at least two treatment cycles, and those with a complete or partial response after two cycles had treatment continued until there was evidence of disease progression, intolerable toxicity, or patient refusal.

### Dose modifications

Toxicity assessment was based on the National Cancer Institute–Common Toxicity Criteria version 2.0. Dose levels and treatment schedule were modified to avoid severe adverse effects. Patients receiving DI had the day-8 docetaxel and irinotecan doses postponed to day 15 if any of the following toxicities was present on day 8: leucocyte count <3000 *μ*l^−1^, platelet count <100 000 *μ*l^−1^ diarrhoea consisting of bloody or watery stools, or increased to two or more diarrhoea within 24 h, abdominal pain rated mild or worse, hepatic toxicity ⩾grade 3, or fever >38°C. If these toxicities occurred on day 15 after skipping the day-8 treatment, DI was stopped in that course.

Patients could receive the next treatment course only if the following criteria were met: leucocyte count ⩾4000 *μ*l^−1^, platelet count ⩾100 000 *μ*l^−1^ AST/ALT <2.0 times the upper limit of normal, total bilirubin ⩽1.5 mg dl^−1^ serum creatinine ⩽ the upper limit of normal, ECOG PS⩽2, neurotoxicity ⩽grade 1, no diarrhoea or oedema. However, if more than 6 weeks passed before these criteria were satisfied, the patient was removed from the study.

Dose modification criteria for each drug are shown in Table 1

**Table 1:** Dose modification criteria

**Toxicities in previous cycle**	**Decrease in docetaxel dose (mg/m^−2^)**	**Decrease in cisplatin dose (mg/m^−2^)**	**Decrease in -irinotecan dose (mg/m^−2^)**
Grade 4 neutropenia lasting ⩾3 days, leucopenia or thrombocytopenia	10	10	10
Grade ⩾2 neurotoxicity	10	10	—
Grade ⩾2 renal toxicity	—	20	—
Grade ⩾2 hepatic toxicity	10	—	5
Grade ⩾3 stomatitis	10	—	—
Grade ⩾2 diarrhoea	—	—	10
Cancellation of day-8 treatment	—	—	10

. If during the previous course, grade 4 leucopenia, grade 4 neutropenia lasting ⩾3 days, or grade 4 thrombocytopenia had occurred, doses of all drugs were reduced by 10 mg m^−2^. Doses of both cisplatin and docetaxel were reduced by 10 mg m^−2^ in subsequent cycles if chemotherapy induced grade ⩾2 neurotoxicity. Moreover, dose of docetaxel was reduced by 10 mg m^−2^ if grade ⩾2 hepatic toxicity or grade ⩾3 stomatitis had occurred. Dose of cisplatin was reduced by 20 /mg/m^2^ if grade ⩾2 renal toxicity occurred. Dose of irinotecan was reduced by 5 mg m^−2^ if grade ⩾2 hepatic toxicity had occurred and by 10 mg m^−2^ if grade ⩾2 diarrhoea or cancellation of day-8 treatment had occurred.

### Evaluation of response and survival

Tumour response was classified according to World Health Organization (WHO) criteria (World Health Organization, 1979). Complete response was defined as complete disappearance of all measurable and assessable disease for at least 4 weeks, Partial response was a ⩾50% decrease in the sum of the products of the two IL largest perpendicular diameters of all measurable tumours lasting at least 4 weeks and without appearance of any new lesions. No change was defined as a <50% decrease or a <25% increase of tumor lesions for at least 4 weeks with no new lesions. Progressive disease was defined as development of new-lesions or a 25% increase in the sum of the products of the two largest perpendicular diameters of all measurable tumors. Duration of response in patients who achieved complete or partial response was measured from the start of treatment to the date of disease progression.

### Statistical methods

Results of this study were evaluated to determine whether the docetaxel plus irinotecan combination warranted further assessment in a phase III trial. Thus, this study was designed to conduct two randomised phase II studies concurrently. We calculated the number of patients required for each of the two studies based on the Fleming's single-stage procedure (Fleming, 1982). In both studies, we set response rates of 40% as target activity level and 20% as the lowest level of interest with a power of 0.9 at a one-sided significance level of 0.05. As a result, a total of 100 qualified patients were to be enrolled, with 50 patients in each treatment arm. The primary objective was to estimate the response rate to both regimens, particularly to irinotecan plus docetaxel.

Overall survival and progression-free survival were analysed by the Kaplan–Meier method. The overall survival was measured from study entry to death. The progression-free survival was measured from study entry until the day of the first evidence of disease progression. If the disease had not progressed by the time of this analysis, progression-free survival was considered censored at the time of the analysis. All comparisons between patient characteristics, response rates, and toxicity incidences were performed by Pearson's *χ*^2^ contingency table analysis.

## RESULTS

### Patient characteristics

From October 1998 to August 1999, 108 patients were assigned to receive DC (*n*=51) or DI (*n*=57). Baseline patient characteristics according to treatment arm are shown in Table 2

**Table 2:** Baseline patient characteristics

		**Docetaxel/cisplatin**	**Docetaxel/irinotecan**	***χ*^2^ text**
No. of patients		51	57	
Gender	Male/female	37/14	38/19	*P*=0.537
Age (years)	Median	62	60	
	Range	39–74	42–77	
PS	0/1	15/36	15/42	*P*=0.830
Histology	Adenocarcinoma	36	44	*P*=0.520
	Squamous cell carcinoma	13	9	
	Others	2	4	
Disease stage	Illb/IV	11/40	14/43	*P*=0.820
Brain metastasis	(+)/(−)	4/47	11/46	*P*=0.086

PS=performance status.

. Patients were well balanced between the two treatment arms in terms of gender, age, performance status, disease stage, and histologic subtypes. There were 23% stage Illb patients and 74% had adenocarcinoma. All patients were included in the survival evaluation, and all were assessable for antitumor efficacy and toxicity.

### Treatment delivery

Patients in both treatment arms received a median of two treatment courses. Two or more courses were delivered to 72.5 and 71.9%, and four courses to 17.6 and 19.1% of patients in the DC and DI arms, respectively. Differences between arms in the number of chemotherapy courses administered were not statistically significant.

### Response to treatment and survival

There were no complete responses. In the DC arm, 19 patients had partial responses for an overall response rate of 37% (Table 3

**Table 3:** Overall response to docetaxel/cisplatin (DC) or docetaxel/irinotecan (DI) in patients with stages IIIb/IV non-small-cell lung cancer

**Response**	**DC (*n*=51) No. pts**	**DI (*n*=67) No. pts**
Complete response	0	0
Partial response	19	18
No change	23	25
Progressive disease	6	14
NE (TRD)	3	0
Response rate	37.3%*	31.6%*
95% Confidence intervals	24.1–51.9%	19.9–45.2%

pts=patients; NE=not evaluable; TRD=treatment-related death.

* *P*=0.55.

). Among DI patients, 18 had partial responses for an overall response rate of 32%. The difference in response rate between arms was not significant (*P*=0.55). Progressive disease was noted in twice as many DI (25%) than DC (12%) patients. Early deaths within 3 months of treatment initiation occurred in 10% (*n*=5) of DC and 5% (*n*=3) of DI patients. The early deaths were treatment-related (three patients, all in the DC arm) or due to disease progression (five patients).

**Figure 2:**
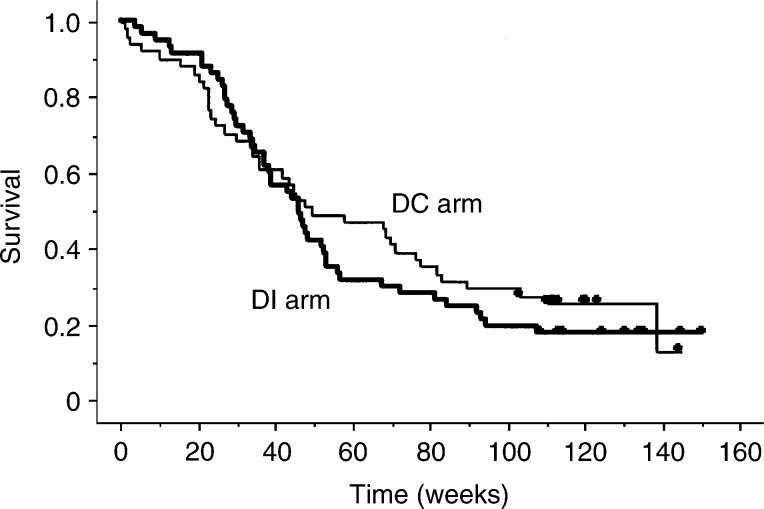
Overall survival according to treatment group, calculated by Kaplan–Meier method. Median survival times were 50 weeks for DC (docetaxel plus cisplatin) and 46 weeks for DI (docetaxel plus irinotecan). *P*=0.50 between treatment groups.

Overall and progression-free survival curves for the two treatment arms are shown in Figures 2

and 3

**Figure 3:**
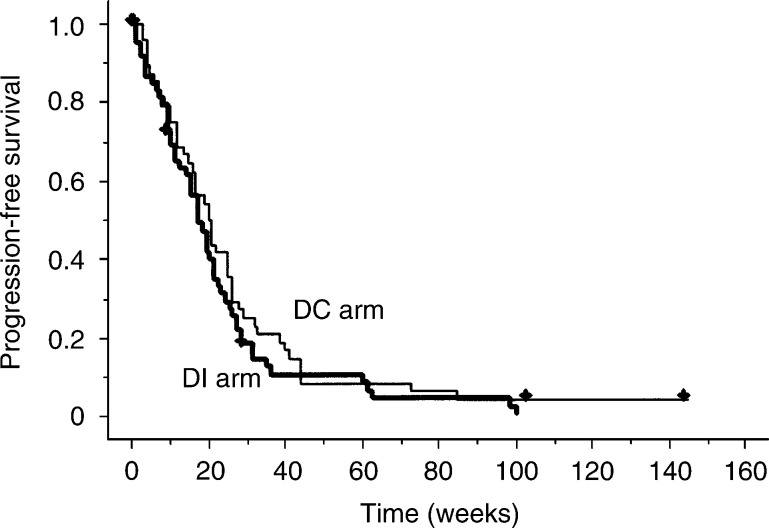
Progression-free survival according to treatment group, calculated by Kaplan–Meier method. Median progression-free survival times were 20 weeks for DC (docetaxel plus cisplatin) and 18 weeks for DI (docetaxel plus irinotecan). *P*=0.33 between treatment groups.

. The median progression-free survival time was 20 weeks (95% confidence interval: 14–25 weeks) in the DC arm *vs* 18 weeks (95% confidence interval: 12–22 weeks) in the DI arm. Median survival times, 1-year survival rates, and 2-year survival rates were 50 weeks (95% confidence interval 34–78 weeks), 47 and 25%, respectively, in the DC arm, and 46 weeks (95% confidence interval: 37–54 weeks), 40 and 18%, respectively, in the DI arm. No significant differences were noted between groups in progression-free survival (*P*=0.33) or overall survival (*P*=0.50), although there were trends toward higher 1-year and 2-year survival rates in the DC.

Second-line chemotherapy was administered to 61 patients (24 DC and 37 DI patients). A total of 22 patients in the DI group received cisplatin-based second-line chemotherapy and five had partial responses to this treatment (overall response rate, 23%). In particular, nine patients were subsequently treated with vinorelbine containing regimen and three patients had a partial response. Only two patients in the DC group received an irinotecan-containing regimen, one of whom had a partial response. Concerning as second-line chest irradiation, 8 patients in the DC group and 13 patients the DI group received.

### Toxicity

Haematologic and nonhaematologic toxicities are listed in Tables 4

**Table 4:** Haematologic toxicity: maximum toxicity grade in any course

	**Docetaxel/cisplatin (% pts)**			**Docetaxel/irinotecan (% pts)**		
**Toxicity/grade**	**2**	**3**	**4**	**2**	**3**	**4**
Leucopenia*	31	43	4	26	40	16
Neutropenia*	10	31	43	4	23	61
Anaemia	47	10	2	46	7	0
Thrombocytopenia**	10	4	0	0	0	0
Febrile neutropenia		20			28	

pts=patients.

* *P*<0.01 for grade 4;

** *P*<0.01 for the sum of grades 2 and 3.

and 5

**Table 5:** Nonhaematologic toxicity: maximum toxicity grade in any course

	**Docetaxel/cisplatin (% pts)**			**Docetaxel/irinotecan (% pts)**		
**Toxicity/grade**	**2**	**3**	**4**	**2**	**3**	**4**
Diarrhoea*	18	6	0	26	12	4
Nausea*	53	33	0	33	18	0
Vomiting**	33	2	4	14	0	0
Peripheral neuropathy	2	0	0	2	0	0
AST increase	8	2	2	7	0	2
ALT increase	14	4	0	9	2	2
ALP increase	8	2	0	4	0	0
Creatinine increase*	10	0	2	0	0	2

pts=patients; AST=aspartate aminotransferase; ALT=alanine aminotransferase; ALP=alkaline phosphatase.

* *P*<0.01 for the sum of grades 2, 3, and 4;

** *P*=0.01 for the sum of grades 2, 3, and 4.

. Grade 4 leucopenia and neutropenia occurred in a significantly higher percentage of DI than DC patients (leucopenia 16 *vs* 4%, *P*<0.01; neutropenia 61 *vs* 43%, *P*<0.01). On the other hand, there was a higher rate of grade ⩾2 thrombocytopenia in the DC than in the DI arm (14 *vs* 0%, *P*<0.01). Rates of anaemia (decrease in haemoglobin) and febrile neutropenia were similar in both groups.

Nonhaematologic toxicities including grade ⩾2 nausea (88 *vs* 51%, *P*<0.01), vomiting (39 *vs* 14%, *P*<0.01), and renal toxicity (increased serum creatinine; 12 *vs* 2%, *P*<0.01) were significantly more prevalent in the DC than in the DI arm, respectively. On the other hand, grade ⩾2 diarrhoea occurred significantly more often in DI than in DC patients (24 *vs* 42%, *P*=0.01). Other nonhaematologic toxicities, such as hepatic toxicity and peripheral neuropathy, were mild and occurred with similar frequency in both groups.

There were three treatment-related deaths in the DC arm, which were due to febrile neutropenia and sepsis (one of these patients also developed perforation of the oesophagus). No treatment-related deaths occurred in the DI arm. The difference in incidence of treatment-related deaths was not significant.

## DISCUSSION

Results of this randomised phase II study showed that the doublet chemotherapy regimens DC and DI had comparable activity in patients with advanced NSCLC. A primary goal of this study was to determine whether the DI combination should be studied in the phase III setting. Although there were no differences between DI and DC–a third-generation cisplatin-containing regimen–in overall and progression-free survival, patients who received DI tended to have lower 1-year and 2-year survival rates. Furthermore, overall toxicity was not reduced in the DI arm compared with the DC arm. Leucopenia and neutropenia were the major toxicities in both groups. As expected, emesis and renal toxicity were more prevalent in patients receiving DC, and diarrhoea occurred more frequently with DI.

Cisplatin has played a prominent role in the treatment of NSCLC, despite a relatively unimpressive single-agent response rate and a relatively severe toxicity profile. In 1995, the Non-Small Cell Lung Cancer Collaborative Group published a pivotal meta-analysis of chemotherapy in lung cancer and demonstrated the advantage of cisplatin-based regimens over best supportive care (Non-Small Cell Lung Cancer Collaborative Group, 1995). In the 1990s, third-generation chemotherapeutic agents, including paclitaxel, docetaxel, vinorelbine, gemcitabine and irinotecan, were shown to have higher response rates often coupled with fewer adverse effects (no renal toxicity, no massive dehydration, less emesis, etc.) than cisplatin. For example, single-agent paclitaxel (Ranson *et al*, 2000), docetaxel (Roszkowski *et al*, 2000), or vinorelbine (The Elderly Lung Cancer Vinorelbine Italian Study Group, 1999) significantly improved survival compared with best supportive care in patients with advanced NSCLC. Studies of single-agent gemcitabine (Perng *et al*, 1997) or irinotecan (Negoro *et al*, 2003) demonstrated a survival benefit comparable to that of second-generation chemotherapy regimens (cisplatin plus vindesine, cisplatin plus etoposide). Based on the above results, we thought that combination chemotherapy consisting of third-generation agents might improve outcome for patients with advanced NSCLC.

Only one published study compared cisplatin-based and noncisplatin-based regimens that included third-generation agents. Georgoulias *et al* (2001) conducted a randomised study of cisplatin plus docetaxel (CD) *vs* gemcitabine plus docetaxel (GD) in 441 advanced NSCLC patients. The noncisplatin regimen provided a comparable response rate (CD 32.4%, GD 30.2%) and median survival time (CD 10 months, GD 9.5 months) but with less toxicity. The authors stated that the non-cisplatin GD regimen would likely be more acceptable to patients based on convenience of administration. However, several randomized trials reported at recent international meetings showed slightly shorter survival times with noncisplatin compared with cisplatin-based combinations. Preliminary results of the EORTC-Lung Cancer Group phase III study of cisplatin plus paclitaxel *vs* cisplatin plus gemcitabine *vs* paclitaxel plus gemcitabine in 480 patients with advanced NSCLC revealed superior overall survival and progression-free survival with the cisplatin-based regimens (Van Meerbeeck *et al*, 2001). Moreover, in a recent Italian–Canadian intergroup study of 501 patients comparing gemcitabine plus vinorelbine with cisplatin plus vinorelbine or gemcitabine, the noncisplatin regimen provided only short-term and sporadic advantages in some quality-of-life components, but there were no significant differences in overall and progression-free survival (Gridelli *et al*, 2002).

The best known noncisplatin platinum-based chemotherapy regimen is the paclitaxel plus carboplatin doublet. A Southwest Oncology Group study compared vinorelbine plus cisplatin with paclitaxel plus carboplatin. No differences in the overall survival or quality of life were noted between the two treatment groups, but toxicity rates were significantly lower in patients who received paclitaxel plus carboplatin (Chen *et al*, 2002). Results of a recent ECOG randomised phase III trial evaluating four platinum-based chemotherapy regimens showed no significant differences in the overall survival, while the paclitaxel plus carboplatin combination was less toxic than cisplatin-based chemotherapy (Schiller *et al*, 2002). Based on these findings, the paclitaxel plus carboplatin regimen is considered a standard therapy for previously untreated patients with advanced NSCLC, with activity comparable to that of cisplatin-based regimens and better tolerability.

The utility of doublet regimens containing third-generation chemotherapeutic agents for advanced NSCLC thus needs to be evaluated against the paclitaxel plus carboplatin combination, and several such studies were reported or are ongoing. The Hellenic Cooperative Oncology Group is conducting a phase III randomised study of paclitaxel plus carboplatin *vs* paclitaxel plus gemcitabine, and final results indicate comparable activity, toxicity and total cost of the two regimens in patients with inoperable NSCLC (Kosmidis *et al*, 2002). The Taiwan group conducted a similar study and found that paclitaxel plus carboplatin and paclitaxel plus gemcitabine had similar efficacy in the treatment of NSCLC, but that paclitaxel plus carboplatin was more cost-effective (Chen *et al*, 2002).

As mentioned in the introductory paragraphs, we conducted a phase I study of docetaxel plus irinotecan (DI) in patients with advanced NSCLC, and had a promising response rate of 48% and median survival time of 48 weeks (Masuda *et al*, 2000). Although we recommended docetaxel 50 mg m^−2^ on day 1 plus irinotecan 50 mg m^−2^ on days 1, 8, and 15 in the phase I study, more than half of patients could not receive irinotecan on day 15 because of haematologic toxicities. Accordingly, the day-15 irinotecan dose was omitted and the day-2 docetaxel dose moved to day 8 and increased from 50 to 60 mg m^−2^ in this randomised phase II trial.

It has been reported that second-line chemotherapy compared with best supportive care may increase the overall survival in patients with advanced NSCLC, and more studies in this regard are needed. In a recent study in which patients received cisplatin-based chemotherapy followed by docetaxel or supportive care alone, the median survival was significantly longer in the docetaxel-treated patients (Shepherd *et al*, 2000). In our study, 52% of patients were treated with second-line chemotherapy. Of these, 19 (33%) DI patients received cisplatin-based second-line chemotherapy, five of whom (26%) responded. Thus, cisplatin-based chemotherapy is capable of exerting antitumour activity in patients who have relapsed after having received noncisplatin-containing regimens.

Only two patients in the DC group received an irinotecan-containing regimen, one of whom had a partial response. As there were only two patients, we cannot judge whether irinotecan-containing regimen is effective for the patients after having received cisplatin-containing regimen.

In conclusion, docetaxel plus irinotecan combinations may be reasonable treatment options for NSCLC patients who cannot tolerate cisplatin. However, as there was no significant difference in the overall survival and no reduction in overall toxicity, DI has not improved on results obtained with DC. Thus, we will not select docetaxel/irinotecan as the experimental regimen in the next phase III study of first-line treatment of advanced NSCLC.
